# Clinical validation and optimization of machine learning models for early prediction of sepsis

**DOI:** 10.3389/fmed.2025.1521660

**Published:** 2025-02-05

**Authors:** Xi Liu, Meiyi Li, Xu Liu, Yuting Luo, Dong Yang, Hui Ouyang, Jiaoling He, Jinyu Xia, Fei Xiao

**Affiliations:** ^1^Department of Infectious Diseases, The Fifth Affiliated Hospital of Sun Yat-sen University, Zhuhai, China; ^2^Guangzhou AID Cloud Technology, Guangzhou, China; ^3^Guangdong Provincial Key Laboratory of Biomedical Imaging, The Fifth Affiliated Hospital, Sun Yat-sen University, Zhuhai, China; ^4^Guangdong Provincial Engineering Research Center of Molecular Imaging, The Fifth Affiliated Hospital, Sun Yat-sen University, Zhuhai, China

**Keywords:** sepsis, machine learning, artificial intelligence, prediction model, infectious disease

## Abstract

**Introduction:**

Sepsis is a global health threat that has a high incidence and mortality rate. Early prediction of sepsis onset can drive effective interventions and improve patients’ outcome.

**Methods:**

Data were collected retrospectively from a cohort of 2,329 adult patients with positive bacteria cultures from a tertiary hospital in China between October 1, 2019 and September 30, 2020. Thirty six clinical features were selected as inputs for the models. We trained models in predicting sepsis by machine learning (ML) methods, including logistic regression, decision tree, random forest (RF), multi-layer perceptron, and light gradient boosting. We evaluated the performance of the five ML models and the evaluation metrics were: area under the ROC curve (AUC), accuracy, F1-score, sensitivity and specificity. The data of another cohort of 2,286 patients between October 1, 2020 and April 1, 2022 were used to validate the performance of the model performing best in the in the internal validation set. Shapley additive explanations (SHAP) method was applied to evaluate feature importance and explain the predictions of this model.

**Results:**

Of the five machine learning models developed, the RF model demonstrated the best performance in terms of AUC (0.818), F1 value (0.38), and sensitivity (0.746). The RF model also has a comparable AUC (0.771) in the external validation set. The SHAP method identified procalcitonin, albumin, prothrombin time, and sex as the important variables contributing to the prediction of sepsis.

**Discussion:**

The RF model we developed showed the greatest potential for early prediction of sepsis in admitted patients, which could aid clinicians in their decision-making process. Our findings also suggested that male patients with bacterial infections and high procalcitonin levels, lower albumin levels, or prolonged prothrombin times were more likely to develop sepsis.

## 1 Introduction

Sepsis is a serious and potentially life-threatening condition characterized by dysregulated host response to severe infection and resultant organ failure (1). It is a leading cause of death from infection (1), with an estimated 48.9 million cases and 11 million deaths worldwide in 2017, accounting for approximately 20% of all global deaths (2). Timely diagnosis and interventions are critical in sepsis management, as sepsis mortality increases significantly with each hour of delay in antimicrobials administration (3). The Sequential organ failure assessment (SOFA) score and quick SOFA are widely used tools for identifying sepsis in clinical practice (4), but they may not be sensitive enough to detect less critical symptoms and may lead to delayed diagnosis and intervention (5) due to the complexity and heterogeneity of the septic population. Therefore, there is an urgent need for more sensitive methods for early identification of sepsis.

Machine learning (ML) method, defined as the field of research that enables computers to learn without explicit programming (6), has been explored for the early predictions of sepsis and the identification of hidden interactions between early signs of the condition (7, 8). Many prediction algorithms have been proposed and successfully used in healthcare, such as decision tree (DT), random forest (RF), multi-layer perceptron (MLP) and light gradient boosting (LGB) (6). The most common approach is to predict sepsis before its onset, typically 6 to 48 h in advance (9, 10). For example, an ensemble algorithm developed by Goh (11) used structured data and unstructured electronic medical records texts to achieve impressive predictive performance 48 h before sepsis onset. Other researchers have focused on real-time prediction using continuous high-frequency physiologic data to predict sepsis earlier than traditional indicators (12, 13). However, many sepsis prediction models have been developed and evaluated using data from patients in intensive care units (ICUs) or emergency departments who have been extensively monitored for various biomarkers, and few models have been tested in real-world settings (14).

In this study, we aimed to use ML method to develop a mathematical model for early prediction of sepsis in admitted patients in real-world settings.

## 2 Materials and methods

### 2.1 Participants and settings

We retrospectively reviewed adult inpatients at the Fifth Affiliated Hospital of Sun Yat-sen University (Zhuhai, P.R. China) admitted to hospital between October 1, 2019 and April 1, 2022. We included patients with pathogen culture-positive and analyzed their electronic records upon admission, converting the data into structured format including age, gender, previous history, vital signs, and laboratory test results (e.g., blood routine, biochemical index, coagulation). Then we excluded patients with false positive culture results defined as contamination or colonization. Patients who were diagnosed as sepsis when they admitted in the hospital would be also excluded.

The development set included 2,329 patients from October 1, 2019 to September 30, 2020. The external validation set included 2,286 patients from October 1, 2020 to April 1, 2022. Patients in both two set had the same inclusion and exclusion criteria.

### 2.2 Definition of sepsis

Sepsis is defined as “Life-threatening organ dysfunction caused by a dysregulated host response to infection, with organ dysfunction being identified as an acute increase in the total SOFA score of two or more points due to infection” (Sepsis-3 definition) (1).

### 2.3 Variable selection

**Figure 1:**
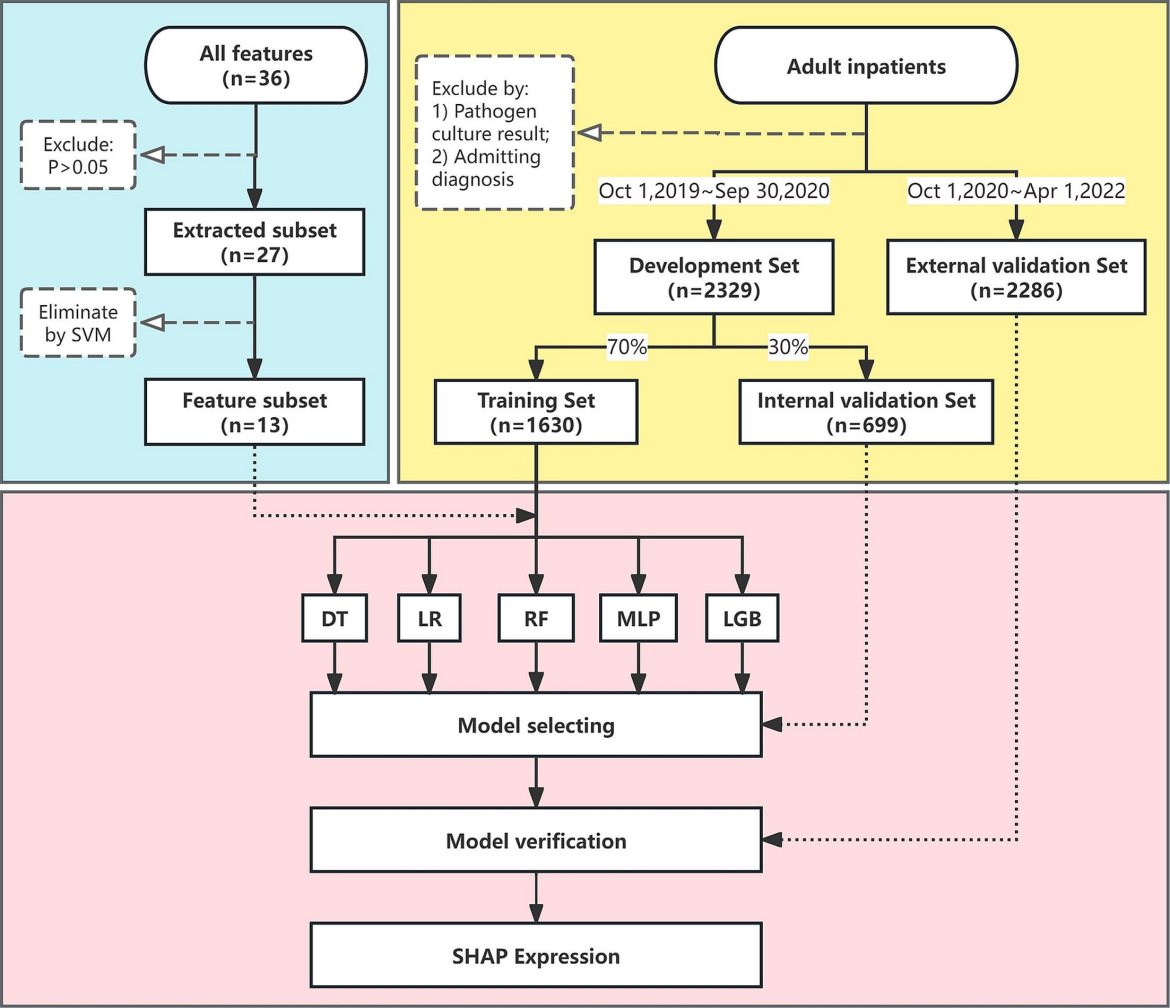
Flow chart of the study. The flow chart illustrates the design and analytic strategy used to train, test, and validate the machine learning algorithms for sepsis prediction. SVM, support vector machine; DT, decision tree; LR, logistic regression; MLP, multi-layer perceptron; LGB, light gradient boosting; SHAP, shapley additive explanations.

The specific selection process is outlined in Figure 1. The authors, including senior physicians (XL and FX) in infectious diseases, held a consensus meeting to identify possible features that might be related to sepsis, and a total of 36 features were selected.

**Figure 2:**
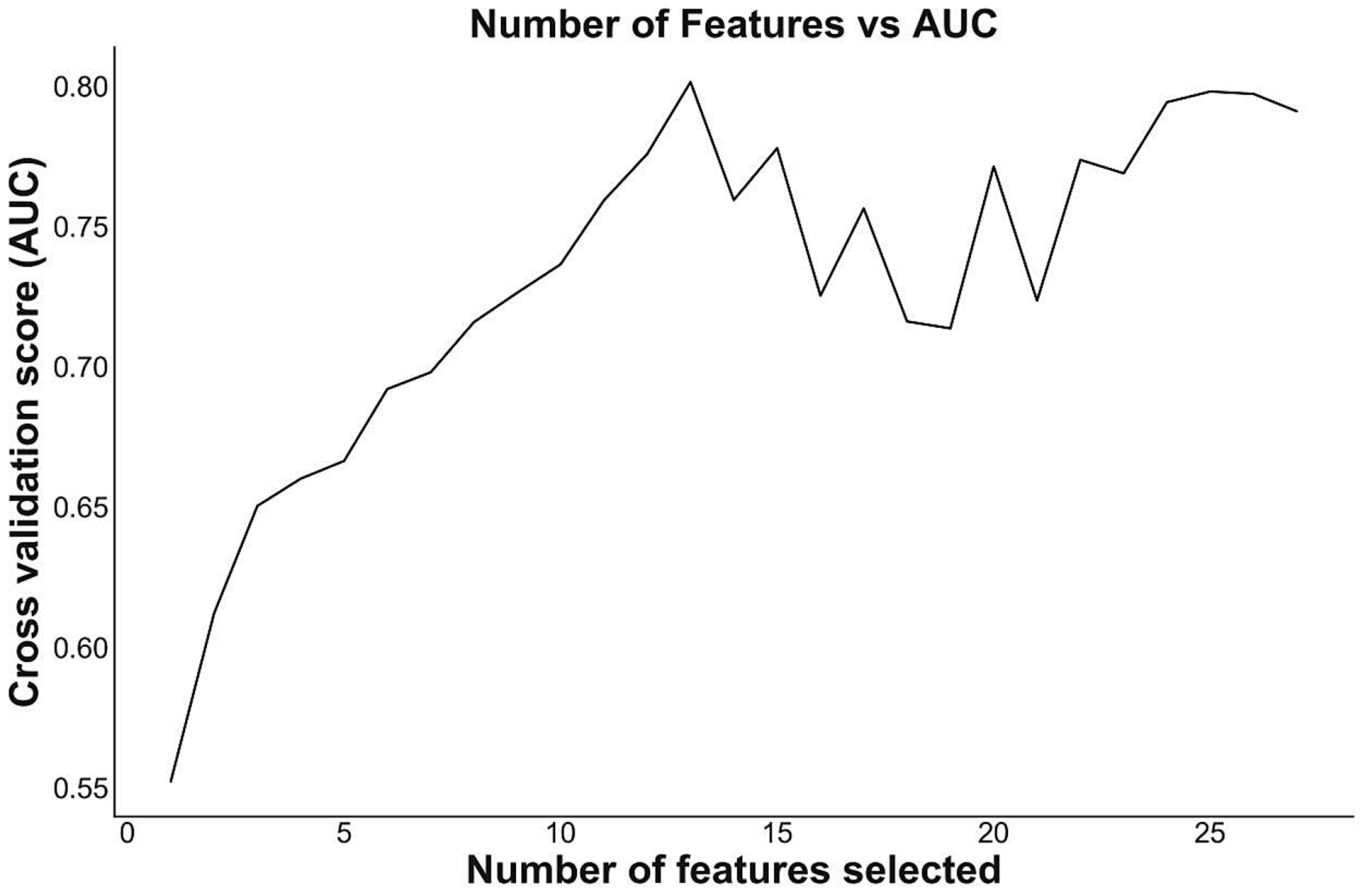
Number of features and AUC. The figure shows the relationship between the number of features and the corresponding AUC. When the number of feature decreases, the cross validation score (AUC) slightly decreases, then increases to a peak, and then drastically goes down. The combination of 13 features had the highest AUC, indicating possible best features to build models.

To reduce the risk of over-fitting, we applied a two-step variable selection procedure. In the first step, we conducted univariate tests to exclude variables that were not significantly related to sepsis (*P >* 0.05, Supplementary Table S1), and 27 variables were remained as extracted subset. In the second step, we used the recursive feature elimination method with support vector machine (SVM) as the base learner to select the best combination of features based on their area under the ROC curve (AUC) value (15) (Figure 2). As a result, a total of 13 variables were ultimately selected as feature subset. The binary classification variables used in the model construction were derived by structuring the original unstructured variables.

Out of the all 36 features, 33 had a missing rate lower than 5%. The remaining 3 features, prothrombin time (PT), lactate dehydrogenase (LDH) and procalcitonin (PCT), had missing rates higher than 10%. Details of the missing variables are shown in Supplementary Table S2. We used the k-Nearest Neighbors (KNN) imputer to predict and fill in missing values. The imputer chose the best fit value based on the KNN algorithm and trends in related columns to fill the missing points (16). Additionally, we conducted necessary statistical hypothesis testing to ensure that there was no statistical significance between the before data imputation and after data imputation (Supplementary Table S2).

### 2.4 Development and evaluation of machine learning models

We used the data of development set to develop models. Among the 2,329 patients in the development set, sepsis occurred in 238 (10.22%) patients. The data sample would be labeled as positive if the patient met the Sepsis-3 definition. To handle sample imbalance and improve prediction accuracy, we used an ensemble method to conduct modeling multiple times with sampled datasets. During each modeling procedure, randomly selected patients without sepsis were included to match the same amount of positive data. Five algorithms were selected as base learners for training: logistic regression (LR), DT, RF and LGB. The LGB model was implemented using the lightgbm (3.2.1) package. The other four models were implemented using the Scikit-learn (0.24.0) package.^1^ To measure the performance of the models, we randomly divided the data of the development set into the training set (70% of the data, 1,630 patients) and the internal validation set (30% of the data, 699 patients). The training set was used to train the models, and the internal validation set was used to evaluate the models. The five trained models were evaluated based on AUC, accuracy, sensitivity, specificity and F1-score. Additionally, we conducted 1,000 times of bootstrap sampling to obtain confidence intervals for these five metrics.

Then we selected the model with best performance in the study, and for further verification, evaluated it using the data of external validation set. The Shapley additive explanations (SHAP) (17) method was also applied on this model in the external validation set to analyze the influence of each feature on the sepsis prediction results during the prediction process. SHAP values have been shown to have high potential for understanding the predictions made by complex ML models (15). SHAP global explanations are based on calculations of the SHAP explanations for all individual patients and averaging them by feature to obtain a cohort view. The larger the mean absolute SHAP value of a feature, the more important that feature is to the model prediction.

### 2.5 Statistical analysis

The pandas (0.25.1) and numpy (1.18.5) packages of Python (Anaconda Distribution, Version 3.7.4) were used for data cleaning. The scipy (1.6.3) package was used for data statistic and examination. For continuous variables, we used mean and standard deviation for statistical description and the Shapiro–Wilk test to test for normal distribution. We used independent samples *t*-test to compare the variables with normal distribution and Mann–Whitney U test for non-normal distribution variables. For categorical variables, we conducted Chi-squared test and Fisher exact test for variables with cell counts less than five.

## 3 Results

### 3.1 Patient characteristics

Supplementary Table S1 showed the demographics, disease history and lab test information of the patients in the study. There were significant differences (*p* < 0.05) in admission white blood cell count, admission neutrophil count, admission neutrophil ratio, admission lymphocyte count, PCT and other indicators between patients with and without sepsis.

### 3.2 Performance of the five ML models

**Figure 3:**
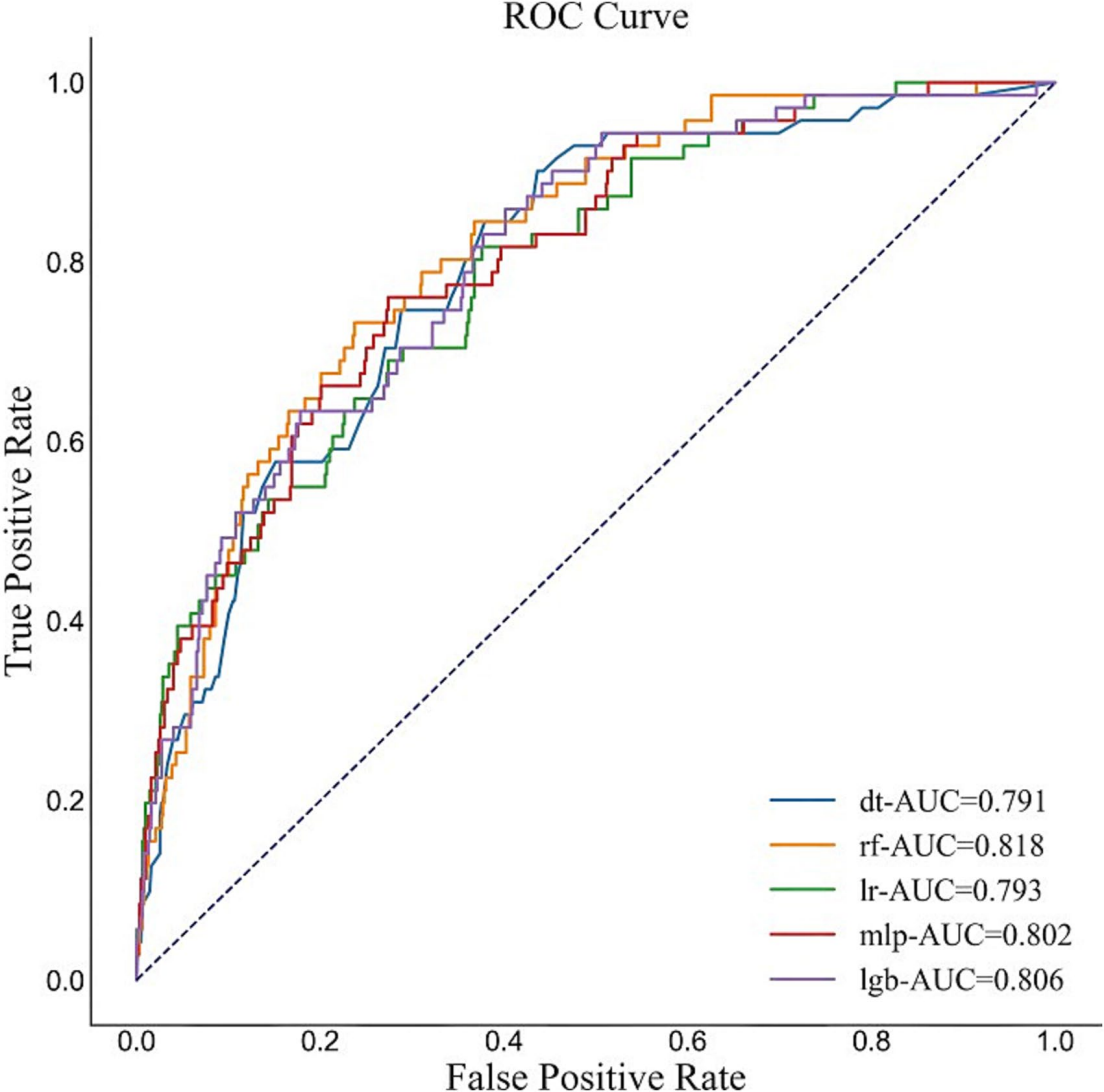
ROC of the five models. The figure shows that the AUC of the RF model is 0.818, which shows the best performance of the five ML models in our study.

The performance of the five models are shown in Supplementary Table S3 and Figure 3. According to the fitting results of the internal validation set, the RF model showed the best performance in terms of AUC (0.818, 95% CI: 0.761–0.862), F1 value (0.38, 95% CI: 0.316–0.447) and sensitivity (0.746, 95% CI: 0.646–0.837) among the five ML models. The accuracy of RF model is 0.753 (95% CI: 0.72–0.78) and the specificity is 0.754 (95% CI: 0.721–0.783).

### 3.3 External validation

**Figure 4:**
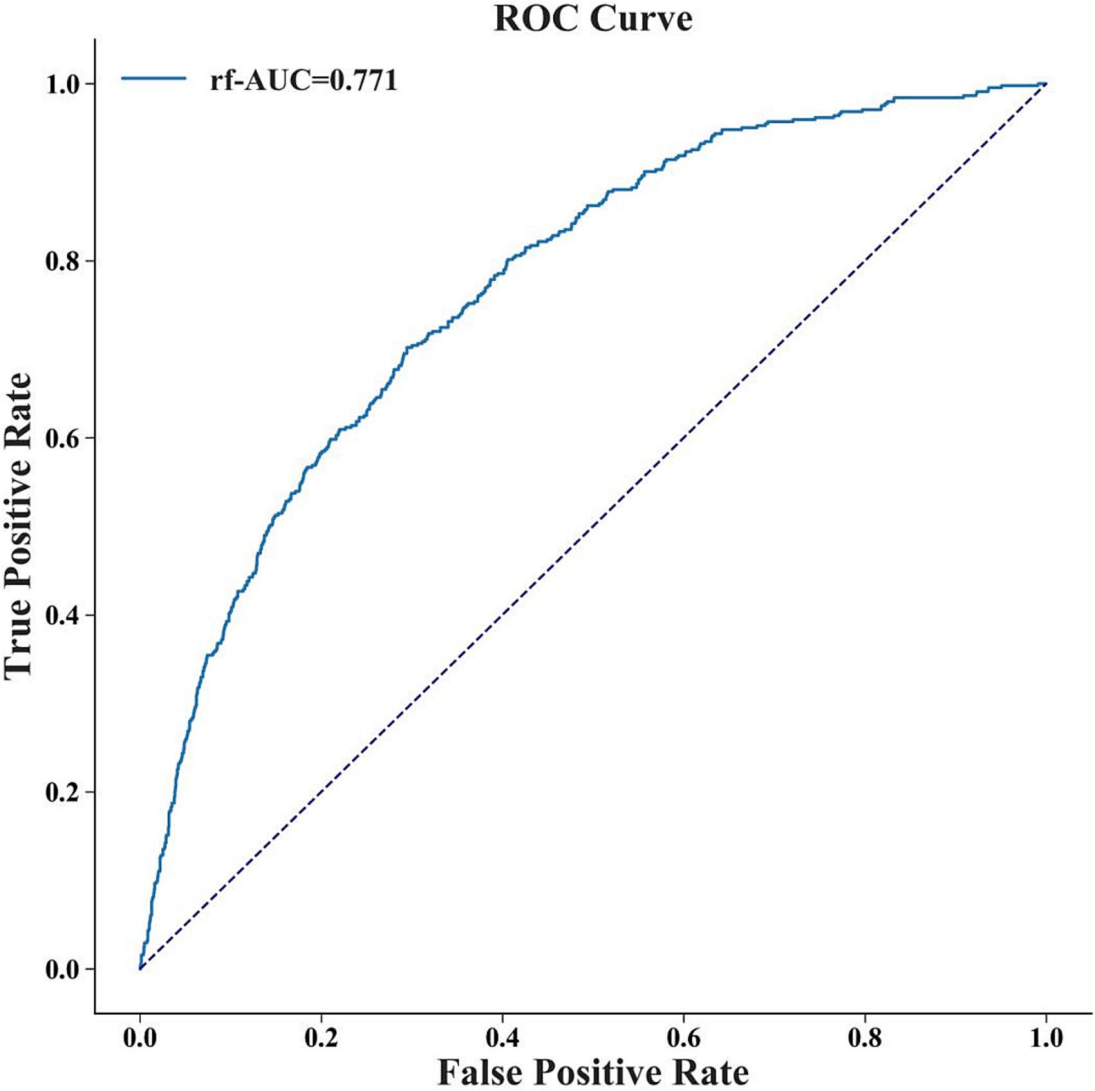
The ROC curve of RF model in external validation set. The AUC of the RF model in the external validation set is 0.771.

To evaluate the RF model, we used a temporal dataset comprising 2,286 patients from October 1, 2020 to April 1, 2022 (Supplementary Table S4). Supplementary Table S5 and Figure 4 showed the performance of the RF model in the external validation set. It had the AUC of 0.771 (95% CI: 0.749–0.790), accuracy of 0.719 (95% CI: 0.704–0.738), F1 value of 0.472 (95% CI: 0.441–0.505), sensitivity of 0.646 (95% CI: 0.607–0.686) and specificity of 0.737 (95% CI: 0.720–0.758). The performance was relatively close but slightly lower, possibly due to differences in the distribution of the training set and the external validation set (Supplementary Table S6).

### 3.4 SHAP values of individual prediction for interpretation

**Figure 5:**
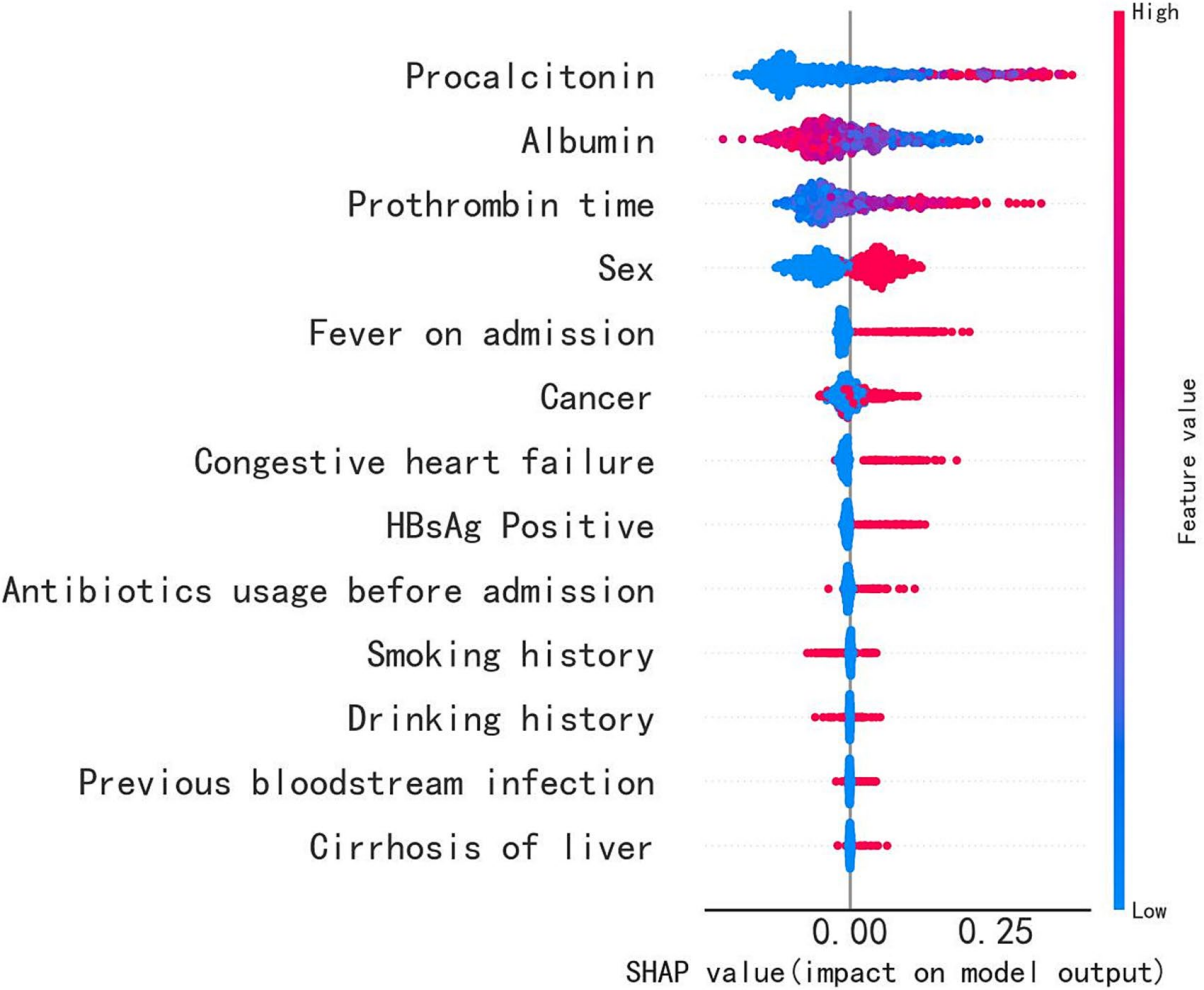
The importance of the feature and the SHAP value. The feature’s position on the Y-axis indicates its importance, and the X-axis represents the SHAP value. The color, ranging from blue to red, represents the feature’s SHAP value from low to high. The violin graph lining up on the midline represents the aggregation of dots representing each case in the internal validation set. The distance between the upper and lower margin of the violin graph represented the number of cases that end up with the same SHAP values provided by this feature. It shows that PCT, albumin, PT and sex are the top four important features.

**Figure 6:**
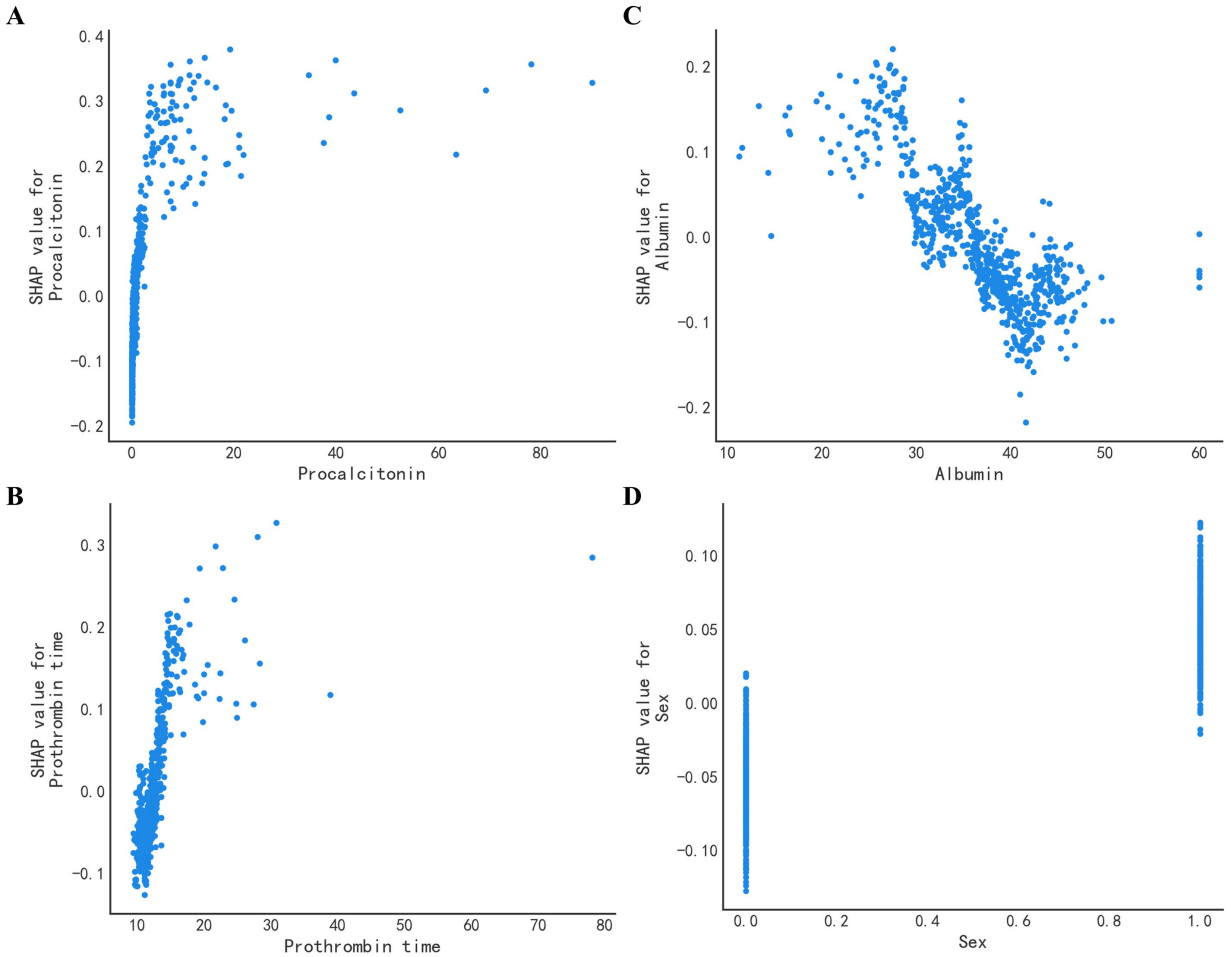
SHAP value of the top four features. The SHAP dependence plot demonstrates the distribution of the SHAP output value of a single feature. **(A)** The SHAP value of PCT. The higher the original PCT value, the higher the corresponding SHAP value. **(B)** The SHAP value of albumin. Opposed to **(A)**, the higher the albumin, the lower the corresponding SHAP value. **(C)** The SHAP value of PT. PT is positively correlated with the SHAP value. **(D)** The SHAP value of Sex. Male is associated with a high SHAP value.

To identify the important features used by the models for predicting sepsis in admitted patients, we computed the feature importance score for all variables. The specific correlation of predictors and sepsis illustrated in the SHAP dependency plot in Figure 5. The SHAP summary plot demonstrated that PCT, albumin, PT and sex were the top four important features. It showed that patients with higher levels of PCT or PT were much more likely to develop sepsis than those with lower levels, while patients with higher levels of albumin were less likely to develop sepsis. Additionally, males were more likely to develop sepsis. When the PCT or PT value grown higher, the corresponding SHAP value also grown higher, indicating a positive correlation (Figures 6A,C). But when albumin value grown higher, the corresponding SHAP value became lower, indicating a negative correlation (Figure 6B). In addition, male was associated with a high SHAP value (Figure 6D).

## 4 Discussion

Reducing the global burden of sepsis is owning important clinical implications. It is crucial for treatment and prognosis to identify and diagnose sepsis as early as possible. To achieve early identification of sepsis, many researchers have applied ML method to the diagnosis of sepsis, and most of these models have analyzed performance indicators such as sensitivity or specificity (16, 18). However, there are still limitations to the use of these ML models in the early identification of sepsis. Some models may input a few of complex laboratory test indicators which are hard to get access in the early admission stage. Additionally, current ML models rely on a large number of available open access datasets and typically analyze a limited number of structured patient variables (14), and these data may come from ICU or emergency department. Besides, it is indispensable to explain the meaning between each feature and the prediction result of the model.

We had noticed these limitation and tried to get over in our study. We developed novel ML models for early prediction of sepsis in the stage of patients’ admitting, using related and accessible features. In addition, we collected both structured and unstructured data from all departments’ inpatients, and analyzed the correlation between these variables and sepsis. Based on its nature of bagging ensemble of large amount of decision trees, RF model performed best among all attempted models. We also conducted external validation and the results showed that our model was effective.

Besides, we also used the SHAP method for analysis and explanation. The SHAP method not only showed the importance of the relationship between variables and sepsis, but also specifically identified positive and negative relationships between variables and sepsis. According to the SHAP chart, PCT, PT, albumin and sex were closely related to sepsis. Interestingly, the SOFA score does not include these four indicators (1). PCT had the strongest correlation with sepsis, indicating a positive correlation. PCT has been widely used to aid in the diagnosis of sepsis (19), in line with our previous understanding. We also noticed that PT was also strongly associated with sepsis. It has been found that PCT and PT are significantly correlated with SOFA score (20), which may be related to microcirculation hypoxia and the resulting microcirculation thrombosis in patients with sepsis. Coagulation dysfunction is very common in patients with sepsis, affecting up to around 80%. In sepsis, inflammation can trigger a coagulation reaction, while the activation of coagulation can further promote an inflammation response (21). Disseminated intravascular coagulation is a condition characterized by the uncontrolled activation of the coagulation cascade, leading to depletion of coagulation factors and the formation of intravascular thrombosis. And this can manifest as prolonged PT (22). In another study, PCT and PT were found to be independent risk factors for sepsis (23). In contrast, a higher albumin level has been found to be inversely related to sepsis, suggesting that a lower albumin level may signal the presence of severe infection (24). The SHAP chart indicated that sex may contribute to a differential risk for developing sepsis, potentially due to differences in estrogen levels (25). However, conflicting evidence exists on the subject (26), and further investigation into sex differences and the mechanisms underlying them is necessary. In conclusion, the results of the SHAP diagram are explicable, and demonstrate the clinical credibility of our model.

Nonetheless, The study has inherent weaknesses. More patient data in multicenter study are needed to evaluate the effectiveness of the proposed system and to make our findings more robust. Besides, there were many cases in which PT and/or PCT values were missing. We tried to delete cases with missing PCT values then conduct the same analysis. It showed that PCT still had the strongest correlation with sepsis, but there were some differences in the result (Supplementary Figure S1, Table S7). Since not all clinicians would perform PT and/or PCT examination on every patient in the real clinical environment, for example, experienced physicians are more inclined to perform the PCT examination, when a patient is highly suspected of bacterial infection. We prefer that the original models (without deletion) may be better adapted to the real clinical settings and achieve the purpose of predicting sepsis. In addition, a prospective cohort study with early antibiotic treatment should be conducted to evaluate whether ML models can improve patient outcomes in the real world.

## 5 Conclusion

In this study, we employed ML method to develop models for early prediction of sepsis in admitted patients and the RF model showed the best performance, which was verified in the external verification set. Our findings also suggested that male patients with bacterial infections and high PCT levels, lower albumin levels, or prolonged PT were more likely to develop sepsis.

## Data Availability

The original contributions presented in the study are included in the article/Supplementary material, further inquiries can be directed to the corresponding authors.

## Footnotes

^1^ https://github.com/scikit-learn/scikit-learn
