# Supplementary material for: Clinical validation and optimization of machine learning models for early prediction of sepsis
Source: Front Med (Lausanne). 2025 Feb 5;12:1521660. doi: 10.3389/fmed.2025.1521660 (PMC11836818; doi:10.3389/fmed.2025.1521660)
Supplement: Supplementary file 1 [file Table_1.docx]

Supplementary Material

**Table1 Variables that were significantly related to sepsis**

| **Variables** | **ALL**  **(n=2329)** | **Non sepsis**  **(n=2091)** | **Sepsis**  **(n=238)** | **Statistics** | **P value** |
| --- | --- | --- | --- | --- | --- |
| Sex | 1181 (50.71%) | 1015 (48.54%) | 166 (69.75%) | 37.602 | <0.001 |
| Age | 59.24±15.47 | 58.89±15.38 | 62.29±15.86 | 10.153 | 0.001 |
| Respiratory diseases | 477 (20.481%) | 421 (20.134%) | 56 (23.529%) | 1.311 | 0.252 |
| Hypertension | 835 (35.852%) | 754 (36.059%) | 81 (34.034%) | 0.298 | 0.585 |
| Coronary heart disease | 215 (9.231%) | 187 (8.943%) | 28 (11.765%) | 1.707 | 0.191 |
| Congestive heart failure | 154 (6.612%) | 126 (6.026%) | 28 (11.765%) | 10.486 | 0.001 |
| Diabetes | 547 (23.486%) | 490 (23.434%) | 57 (23.95%) | 0.009 | 0.923 |
| Renal failure | 242 (10.391%) | 202 (9.66%) | 40 (16.807%) | 10.965 | 0.001 |
| Fatty liver | 148 (6.355%) | 139 (6.648%) | 9 (3.782%) | 2.488 | 0.115 |
| Cirrhosis of liver | 59 (2.533%) | 45 (2.152%) | 14 (5.882%) | 10.579 | 0.001 |
| Autoimmune diseases | 121 (5.195%) | 110 (5.261%) | 11 (4.622%) | 0.071 | 0.79 |
| Transplantation recipients | 31 (1.331%) | 26 (1.243%) | 5 (2.101%) | 0.632 | 0.426 |
| Cancer | 697 (29.927%) | 608 (29.077%) | 89 (37.395%) | 6.659 | 0.01 |
| HBsAg Positive | 181 (7.772%) | 151 (7.221%) | 30 (12.605%) | 7.906 | 0.005 |
| HIV infection | 24 (1.03%) | 23 (1.1%) | 1 (0.42%) | 0.416 | 0.519 |
| Glucocorticoids or immunosuppressant usage | 154 (6.612%) | 136 (6.504%) | 18 (7.563%) | 0.235 | 0.627 |
| Smoking history | 354 (15.2%) | 307 (14.682%) | 47 (19.748%) | 3.871 | 0.049 |
| Drinking history | 171 (7.342%) | 144 (6.887%) | 27 (11.345%) | 5.604 | 0.018 |
| Fever on admission | 257 (11.035%) | 189 (9.039%) | 68 (28.571%) | 81.065 | <0.001 |
| Previous bloodstream infection | 87 (3.736%) | 67 (3.204%) | 20 (8.403%) | 14.649 | <0.001 |
| Antibiotics usage before admission | 135 (5.796%) | 101 (4.83%) | 34 (14.286%) | 33.276 | <0.001 |
| White blood cell count | 9.07±7.17 | 8.64±5.9 | 12.85±13.47 | 36.342 | <0.001 |
| Neutrophil count | 7.1±22.95 | 6.75±24.03 | 10.24±8.4 | 58.878 | <0.001 |
| Neutrophil ratio | 69.69±15.6 | 68.63±14.99 | 78.96±17.62 | 127.56 | <0.001 |
| Lymphocyte count | 1.54±1.5 | 1.58±1.48 | 1.12±1.65 | 127.352 | <0.001 |
| Blood platelet count | 238.01±107.14 | 242.71±103.55 | 196.72±127.62 | 51.151 | <0.001 |
| Hemoglobin | 116.18±37.81 | 117.77±38.29 | 102.14±29.88 | 69.574 | <0.001 |
| Procalcitonin | 2.73±10.08 | 1.75±7.69 | 11.32±19.86 | 212.281 | <0.001 |
| Prothrombin time | 12.46±4.12 | 12.23±3.45 | 14.48±7.58 | 117.979 | <0.001 |
| Urea | 11.03±140.54 | 11.12±148.3 | 10.16±7.71 | 65.264 | <0.001 |
| Serum creatinine | 114.66±158.31 | 107.88±147.69 | 174.29±223.3 | 48.504 | <0.001 |
| Alanine aminotransferase | 29.19±86.18 | 26.43±80.47 | 53.42±123.27 | 17.548 | <0.001 |
| Aspartate aminotransferase | 33.5±57.9 | 28.94±42.56 | 73.61±123.11 | 84.406 | <0.001 |
| Total bilirubin | 14.75±34.54 | 12.38±21.63 | 35.58±84.32 | 25.565 | <0.001 |
| Albumin | 36.56±6.78 | 37.12±6.44 | 31.57±7.58 | 131.101 | <0.001 |
| Lactic dehydrogenase | 223.37±222.11 | 208.88±137.08 | 350.67±548.42 | 77.809 | <0.001 |

**Table 2 The data before and after input**

| **Variables** | **Subtype** | **Pre-fill** | **Post-fill** | **P values** | **Missing Percentage** |
| --- | --- | --- | --- | --- | --- |
| Sex | 0 | 1148 (49.29%) | 1148 (49.29%) | 1.000 | 0.000 |
|  | 1 | 1181 (50.71%) | 1181 (50.71%) | 1.000 | 0.000 |
| Age | - | 59.24±15.47 | 59.24±15.47 | 1.000 | 0.000 |
| Respiratory diseases | 0 | 1851 (79.51%) | 1852 (79.52%) | 0.996 | 0.000 |
|  | 1 | 477 (20.49%) | 477 (20.48%) | 0.996 | 0.000 |
| Hypertension | 0 | 1493 (64.13%) | 1494 (64.15%) | 0.994 | 0.000 |
|  | 1 | 835 (35.87%) | 835 (35.85%) | 0.994 | 0.000 |
| Coronary heart disease | 0 | 2113 (90.76%) | 2114 (90.77%) | 0.997 | 0.000 |
|  | 1 | 215 (9.24%) | 215 (9.23%) | 0.997 | 0.000 |
| Congestive heart failure | 0 | 2174 (93.38%) | 2175 (93.39%) | 0.998 | 0.000 |
|  | 1 | 154 (6.62%) | 154 (6.61%) | 0.998 | 0.000 |
| Diabetes | 0 | 1781 (76.5%) | 1782 (76.51%) | 0.995 | 0.000 |
|  | 1 | 547 (23.5%) | 547 (23.49%) | 0.995 | 0.000 |
| Renal failure | 0 | 2086 (89.6%) | 2087 (89.61%) | 0.997 | 0.000 |
|  | 1 | 242 (10.4%) | 242 (10.39%) | 0.997 | 0.000 |
| Fatty liver | 0 | 2180 (93.64%) | 2181 (93.65%) | 0.998 | 0.000 |
|  | 1 | 148 (6.36%) | 148 (6.35%) | 0.998 | 0.000 |
| Cirrhosis of liver | 0 | 2269 (97.47%) | 2270 (97.47%) | 0.999 | 0.000 |
|  | 1 | 59 (2.53%) | 59 (2.53%) | 0.999 | 0.000 |
| Autoimmune diseases | 0 | 2207 (94.8%) | 2208 (94.8%) | 0.998 | 0.000 |
|  | 1 | 121 (5.2%) | 121 (5.2%) | 0.998 | 0.000 |
| Transplantation recipients | 0 | 2297 (98.67%) | 2298 (98.67%) | 0.999 | 0.000 |
|  | 1 | 31 (1.33%) | 31 (1.33%) | 0.999 | 0.000 |
| Cancer | 0 | 1632 (70.1%) | 1632 (70.07%) | 0.987 | 0.000 |
|  | 1 | 696 (29.9%) | 697 (29.93%) | 0.987 | 0.000 |
| HBsAg Positive | 0 | 2147 (92.26%) | 2148 (92.23%) | 0.974 | 0.001 |
|  | 1 | 180 (7.74%) | 181 (7.77%) | 0.974 | 0.001 |
| HIV infection | 0 | 2303 (98.97%) | 2305 (98.97%) | 0.998 | 0.001 |
|  | 1 | 24 (1.03%) | 24 (1.03%) | 0.998 | 0.001 |
| Previous bloodstream infection | 0 | 2241 (96.26%) | 2242 (96.26%) | 0.998 | 0.000 |
|  | 1 | 87 (3.74%) | 87 (3.74%) | 0.998 | 0.000 |
| Smoking history | 0 | 1973 (84.79%) | 1975 (84.8%) | 0.993 | 0.001 |
|  | 1 | 354 (15.21%) | 354 (15.2%) | 0.993 | 0.001 |
| Drinking history | 0 | 2157 (92.65%) | 2158 (92.66%) | 0.998 | 0.000 |
|  | 1 | 171 (7.35%) | 171 (7.34%) | 0.998 | 0.000 |
| Fever on admission | 0 | 2070 (88.96%) | 2072 (88.97%) | 0.994 | 0.001 |
|  | 1 | 257 (11.04%) | 257 (11.03%) | 0.994 | 0.001 |
| Antibiotics usage before admission | 0 | 2176 (94.2%) | 2194 (94.2%) | 0.996 | 0.008 |
|  | 1 | 134 (5.8%) | 135 (5.8%) | 0.996 | 0.008 |
| Using hormone or immunosuppressant | 0 | 2174 (93.38%) | 2175 (93.39%) | 0.998 | 0.000 |
|  | 1 | 154 (6.62%) | 154 (6.61%) | 0.998 | 0.000 |
| White blood cell count | - | 9.08±7.17 | 9.07±7.17 | 1.000 | 0.003 |
| Neutrophil count | - | 7.11±23.0 | 7.1±22.95 | 1.000 | 0.004 |
| Neutrophil ratio | - | 69.71±15.61 | 69.69±15.6 | 1.000 | 0.003 |
| Lymphocyte count | - | 1.54±1.5 | 1.54±1.5 | 1.000 | 0.003 |
| Blood platelet count | - | 237.95±107.26 | 238.01±107.14 | 1.000 | 0.003 |
| Hemoglobin | - | 116.14±37.86 | 116.18±37.81 | 1.000 | 0.004 |
| Procalcitonin | - | 3.28±11.93 | 2.73±10.08 | 0.096 | 0.375 |
| Prothrombin time | - | 12.53±4.32 | 12.46±4.12 | 0.899 | 0.100 |
| Urea | - | 9.85±127.72 | 11.03±140.54 | 1.000 | 0.029 |
| Serum creatinine | - | 115.28±159.88 | 114.66±158.31 | 1.000 | 0.025 |
| Alanine aminotransferase | - | 29.4±87.57 | 29.19±86.18 | 1.000 | 0.033 |
| Aspartate aminotransferase | - | 33.79±58.69 | 33.5±57.9 | 1.000 | 0.028 |
| Total bilirubin | - | 14.95±35.15 | 14.75±34.54 | 1.000 | 0.038 |
| Albumin | - | 36.48±6.84 | 36.56±6.78 | 1.000 | 0.040 |
| Lactic dehydrogenase | - | 232.95±252.56 | 223.37±222.11 | 0.624 | 0.251 |

**Table 3 Performance of the five ML models**

| **Model** | **AUC** | **Accuracy** | **F1** | **Sensitivity** | **Specificity** |
| --- | --- | --- | --- | --- | --- |
| DT | 0.791 (0.746, 0.844) | 0.708 (0.677, 0.739) | 0.340 (0.275, 0.407) | 0.743 (0.641, 0.835) | 0.704 (0.67, 0.736) |
| RF | 0.818 (0.761, 0.862) | 0.753 (0.72, 0.78) | 0.380 (0.316, 0.447) | 0.746 (0.646, 0.837) | 0.754 (0.721, 0.783) |
| LR | 0.793 (0.736, 0.845) | 0.752 (0.715, 0.783) | 0.337 (0.27, 0.417) | 0.627 (0.534, 0.733) | 0.766 (0.73, 0.796) |
| MLP | 0.802 (0.743, 0.847) | 0.774 (0.74, 0.807) | 0.368 (0.295, 0.444) | 0.648 (0.537, 0.746) | 0.788 (0.756, 0.822) |
| LGB | 0.806 (0.758, 0.861) | 0.732 (0.698, 0.764) | 0.337 (0.279, 0.404) | 0.676 (0.576, 0.799) | 0.738 (0.702, 0.77) |

**Table 4 Clinical characters of the external validation set**

| **Variables** | **ALL**  **(n=2286)** | **Non sepsis**  **(n=1843)** | **Sepsis**  **(n=443)** | **Statistics** | **P value** |
| --- | --- | --- | --- | --- | --- |
| Sex (Female) | 1251 (54.724%) | 976 (52.957%) | 275 (62.077%) | 11.623 | 0.001 |
| Age | 60.26±16.41 | 59.62±16.46 | 62.89±15.92 | 14.05 | <0.001 |
| Respiratory diseases | 293 (12.817%) | 259 (14.053%) | 34 (7.675%) | 12.438 | <0.001 |
| Hypertension | 917 (40.114%) | 728 (39.501%) | 189 (42.664%) | 1.359 | 0.244 |
| Coronary heart disease | 187 (8.18%) | 144 (7.813%) | 43 (9.707%) | 1.462 | 0.227 |
| Congestive heart failure | 200 (8.749%) | 124 (6.728%) | 76 (17.156%) | 47.347 | <0.001 |
| Diabetes | 588 (25.722%) | 467 (25.339%) | 121 (27.314%) | 0.629 | 0.428 |
| Renal failure | 334 (14.611%) | 275 (14.921%) | 59 (13.318%) | 0.613 | 0.434 |
| Fatty liver | 195 (8.53%) | 160 (8.681%) | 35 (7.901%) | 0.188 | 0.665 |
| Cirrhosis of liver | 65 (2.843%) | 48 (2.604%) | 17 (3.837%) | 1.545 | 0.214 |
| Autoimmune diseases | 86 (3.762%) | 68 (3.69%) | 18 (4.063%) | 0.054 | 0.817 |
| Transplantation recipients | 31 (1.356%) | 13 (0.705%) | 18 (4.063%) | 27.646 | <0.001 |
| Cancer | 563 (24.628%) | 403 (21.867%) | 160 (36.117%) | 38.311 | <0.001 |
| HBsAg Positive | 165 (7.218%) | 127 (6.891%) | 38 (8.578%) | 1.276 | 0.259 |
| HIV infection | 22 (0.962%) | 14 (0.76%) | 8 (1.806%) | 3.077 | 0.079 |
| Glucocorticoids or immunosuppressant usage | 167 (7.305%) | 113 (6.131%) | 54 (12.19%) | 18.474 | <0.001 |
| Smoking history | 507 (22.178%) | 421 (22.843%) | 86 (19.413%) | 2.24 | 0.134 |
| Drinking history | 237 (10.367%) | 191 (10.364%) | 46 (10.384%) | 0.006 | 0.941 |
| Fever on admission | 519 (22.703%) | 387 (20.998%) | 132 (29.797%) | 15.258 | <0.001 |
| Previous bloodstream infection | 27 (1.181%) | 18 (0.977%) | 9 (2.032%) | 2.562 | 0.109 |
| Antibiotics usage before admission | 704 (30.796%) | 582 (31.579%) | 122 (27.54%) | 2.548 | 0.110 |
| White blood cell count | 87.97±1842.63 | 99.01±2039.01 | 42.04±472.85 | 8.208 | 0.004 |
| Neutrophil count | 7.05±4.25 | 6.77±3.62 | 8.23±6.1 | 40.974 | <0.001 |
| Neutrophil ratio | 0.82±1.05 | 0.81±1.14 | 0.88±0.51 | 64.079 | <0.001 |
| Lymphocyte count | 1.75±1.56 | 1.74±0.82 | 1.8±3.12 | 46.615 | <0.001 |
| Blood platelet count | 276.66±147.35 | 308.65±132.03 | 143.58±132.59 | 513.618 | <0.001 |
| Hemoglobin | 112.78±24.45 | 116.21±23.39 | 98.5±23.58 | 200.019 | <0.001 |
| Procalcitonin | 3.83±14.86 | 1.65±8.16 | 12.89±27.6 | 313.872 | <0.001 |
| Prothrombin time | 14.35±7.67 | 13.5±6.55 | 17.87±10.47 | 234.446 | <0.001 |
| Urea | 8.23±7.16 | 7.27±5.81 | 12.23±10.22 | 236.975 | <0.001 |
| Serum creatinine | 139.04±182.38 | 128.3±176.42 | 183.7±199.45 | 107.769 | <0.001 |
| Alanine aminotransferase | 38.22±169.49 | 27.72±58.6 | 81.89±363.09 | 36.491 | <0.001 |
| Aspartate aminotransferase | 66.48±436.43 | 32.32±53.21 | 208.62±973.54 | 144.604 | <0.001 |
| Total bilirubin | 14.57±33.97 | 10.06±15.81 | 33.34±66.96 | 127.306 | <0.001 |
| Albumin | 37.44±5.52 | 37.95±5.24 | 35.33±6.14 | 71.389 | <0.001 |
| Lactic dehydrogenase | 306.02±797.72 | 253.44±636.4 | 524.77±1241.95 | 130.186 | <0.001 |

**Table 5 Performance of the RF model in the external validation set**

| **AUC** | **Accuracy** | **F1** | **Sensitivity** | **Specificity** |
| --- | --- | --- | --- | --- |
| 0.771 (0.749-0.790) | 0.719 (0.704-0.738) | 0.472 (0.441-0.505) | 0.646 (0.607-0.686) | 0.737 (0.720-0.758) |

**Table 6 Differences between distribution of the training set and the external validation set**

| **Characteristics** | **Subtype** | **Training set** | **External validation set** | ***P* value** |
| --- | --- | --- | --- | --- |
| Sex | 0 | 814 (49.94%) | 1251 (54.72%) | 0.024 |
|  | 1 | 816 (50.06%) | 1035 (45.28%) |  |
| Congestive heart failure | 0 | 1533 (94.05%) | 2086 (91.25%) | 0.013 |
|  | 1 | 97 (5.95%) | 200 (8.75%) |  |
| Cirrhosis of liver | 0 | 1590 (97.55%) | 2221 (97.16%) | 0.570 |
|  | 1 | 40 (2.45%) | 65 (2.84%) |  |
| Cancer | 0 | 1155 (70.86%) | 1723 (75.37%) | 0.016 |
|  | 1 | 475 (29.14%) | 563 (24.63%) |  |
| HBsAg Positive | 0 | 1502 (92.15%) | 2121 (92.78%) | 0.570 |
|  | 1 | 128 (7.85%) | 165 (7.22%) |  |
| Smoking history | 0 | 1376 (84.42%) | 1779 (77.82%) | <0.001 |
|  | 1 | 254 (15.58%) | 507 (22.18%) |  |
| Drinking history | 0 | 1511 (92.7%) | 2049 (89.63%) | 0.012 |
|  | 1 | 119 (7.3%) | 237 (10.37%) |  |
| Fever on admission | 0 | 1452 (89.08%) | 1767 (77.3%) | <0.001 |
|  | 1 | 178 (10.92%) | 519 (22.7%) |  |
| Previous bloodstream infection | 0 | 1569 (96.26%) | 2259 (98.82%) | <0.001 |
|  | 1 | 61 (3.74%) | 27 (1.18%) |  |
| Antibiotics usage before admission | 0 | 1534 (94.11%) | 1582 (69.2%) | <0.001 |
|  | 1 | 96 (5.89%) | 704 (30.8%) |  |
| Procalcitonin | - | 2.92±10.82 | 3.83±14.86 | <0.001 |
| Prothrombin time | - | 12.47±4.32 | 14.35±7.67 | <0.001 |
| Albumin | - | 36.59±6.83 | 37.44±5.52 | <0.001 |


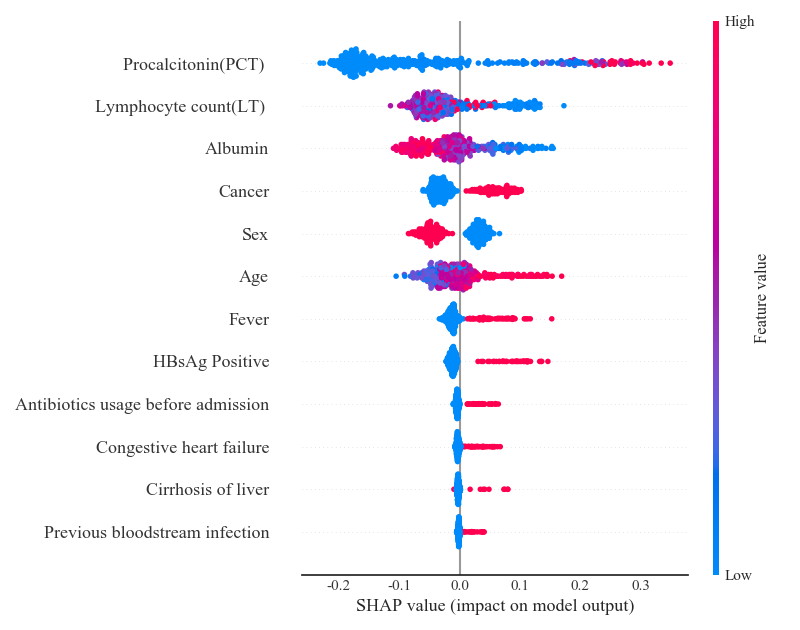


**Supplementary Figures S1. SHAP value of the additional model.** Cases with with missing PCT values were deleted. Then the model was conducted the same analysis.

**Table 7 Performance of the models after deletion**

| **Model** | **AUC** | **Accuracy** | **F1** | **Sensitivity** | **Specificity** |
| --- | --- | --- | --- | --- | --- |
| DT | 0.786(0.726, 0.841) | 0.707(0.665, 0.743) | 0.412(0.32, 0.484) | 0.704(0.581, 0.81) | 0.708(0.661, 0.747) |
| RF | 0.821(0.758, 0.877) | 0.757(0.709, 0.795) | 0.456(0.369, 0.539) | 0.718(0.602, 0.836) | 0.764(0.719, 0.801) |
| LR | 0.787(0.724, 0.841) | 0.72(0.676, 0.762) | 0.398(0.312, 0.475) | 0.652(0.519, 0.756) | 0.732(0.684, 0.78) |
| MLP | 0.809(0.741, 0.867) | 0.767(0.73, 0.804) | 0.461(0.362, 0.54) | 0.679(0.582, 0.779) | 0.782(0.746, 0.823) |
| LGB | 0.8(0.731, 0.864) | 0.739(0.686, 0.783) | 0.447(0.361, 0.527) | 0.731(0.62, 0.834) | 0.741(0.691, 0.792) |
